# Effect of short-term blood pressure variability on functional outcome after intra-arterial treatment in acute stroke patients with large-vessel occlusion

**DOI:** 10.1186/s12883-019-1457-5

**Published:** 2019-09-26

**Authors:** Tianli Zhang, Xiaolong Wang, Chao Wen, Feng Zhou, Shengwei Gao, Xiaodong Zhang, Shiqin Lin, Jing Shi, Weirong Li

**Affiliations:** Department of Neurology, Taiyuan Central Hospital, Taiyuan city, 030009 Shanxi Province China

**Keywords:** Blood pressure, Acute ischemic stroke, Large vessel occlusion, Functional outcome

## Abstract

**Background:**

Endovascular treatment (EVT) is advocated for acute ischaemic stroke with large-vessel occlusion (LVO), but perioperative periods are challenging. This study investigated the relationship between post-EVT short-term blood pressure variability (BPV) and early outcomes in LVO patients.

**Methods:**

We retrospectively reviewed 72 LVO patients undergoing EVT between June 2015 and June 2018. Hourly systolic and diastolic blood pressures (SBP and DBP, respectively) were recorded in the first 24 h post-EVT. BPV were evaluated as standard deviation (SD), coefficient of variation (CV), and successive variation (SV) separately for SBP and DBP. Functional independence at 3 months was defined as a modified Rankin Scale (mRS) score of 0–2.

**Results:**

For 58.3% patients with favorable outcomes, the median National Institutes of Health Stroke Scale and Alberta Stroke Program Early CT scores on admission were 14 and 8, respectively. The maximum SBP ([154.3 ± 16.8] vs. [163.5 ± 15.6], *P* = 0.02), systolic CV ([8. 8% ± 2.0%] vs. [11.0% ± 1.8], *P* < 0.001), SV ([11.4 ± 2.3] vs. [14.6 ± 2.0], *P* < 0.001), and SD ([10.5 ± 2.4] vs. [13.8 ± 3.9], *P* < 0.001) were lower in patients with favorable outcomes. On multivariable logistic regression analysis, systolic SV (OR: 4.273, 95% CI: 1.030 to 17.727, *P* = 0.045) independently predicted unfavorable prognosis. The area under the curve was 0.868 (95% CI: 0.781 to 0.955, *P* < 0.001), and sensitivity and specificity were 93.3% and 73.8%, respectively, showing excellent predictive value for 3-month poor-outcomes.

**Conclusions:**

Decreased systolic SV following intra-arterial therapies result in favorable outcomes at 3 months. Systolic SV may be a novel predictor of functional prognosis in LVO patients.

**Supplementary information:**

**Supplementary information** accompanies this paper at 10.1186/s12883-019-1457-5.

## Background

Early endovascular treatment (EVT) for patients who had acute ischaemic stroke with large-vessel occlusion (AIS-LVO) is highly recommended based on the findings of six randomized controlled clinical trials [1–6]_._ However, several factors during the perioperative period of EVT, including blood pressure (BP) management, need urgent attention. The optimal range of BP following EVT remains unclear. The 2018 American Heart Association and American Stroke Association guidelines for the early management of patients with AIS recommends maintaining the BP at < 180/105 mmHg (IIb, B-NR) in patients who underwent mechanical thrombectomy (MT) with successful reperfusion [7]. The 2018 Chinese guidelines also recommend a target BP of 140/90 or a BP of 20 mmHg lower than that at baseline, but it should also not be less than 100/60 mmHg (II, C) [8]. However, reperfusion injury may occur dispite maintaining the BP within the target range.

Blood pressure variability (BPV) is the fluctuation of BP over a certain period of time. In the acute stage of cerebrovascular disease, the fluctuation of cerebral perfusion pressure is aggravated by short-term BPV due to impaired automatic regulation of cerebral blood flow [9]. Hypertension during the perioperative period may lead to adverse events such as the reperfusion syndrome and cardiovascular complications, while hypotension may lead to hypoperfusion and increases the risk of infarction. A recent systematic review reported that increased BPV after stroke is associated with higher rates of intracranial haemorrhage and disability [10]. However, there is limited epidemiological evidence to evaluate the relationship between BP level and early functional prognosis after EVT. Thus, this study aimed to explore the association between short-term BPV in the first 24 h following EVT and functional outcomes in patients with AIS-LVO.

## Methods

### Patient selection

This is a retrospective study was approved by the Institutional Review Board of Taiyuan Central Hospital, Shanxi, People’s Republic of China. Consecutive AIS-LVO patients who underwent emergency EVT in the tertiary care stroke center of Taiyuan Central Hospital between June 2015 and June 2018 were enrolled. The inclusion criteria were as follows: (1) age of ≥18 years; (2) AIS confirmed via head computed tomography (CT) or magnetic resonance imaging at admission; (3) occlusion of the internal carotid artery or M1 of the middle cerebral artery diagnosed within 6 h after onset by digital subtraction angiography; (4) preoperative Alberta Stroke Program Early CT Score (ASPECTS) of ≥6, prestroke modified Rankin Scale (mRS) score of < 2, and National Institutes of Health Stroke Scale (NIHSS) score of ≥6; (5) treatment initiated (groin puncture) within 6 h of symptom onset; (6) clinical features and BP recorded at baseline and hourly for at least 24 h after EVT; and (7) follow up by phone or face-to-face consultations at 3 months with complete documentation. Patients were excluded if they had active bleeding or a bleeding tendency (including primary intracerebral haemorrhage, and potential causes such as gastrointestinal malignancy, liver cirrhosis, renal failure, hematologic tumour, vitamin K deficiency, and sepsis, which could lead to bleeding events), serious heart failure or respiratory failure pre-admission, glucose < 50 mg/dL or > 400 mg/dL, severe hypertension beyond drug control, and severe non-cardiovascular events that occurred within 3 months of follow-up. The management of patients with AIS-LVO was based on the Chinese guidelines for diagnosis and treatment of AIS 2014 and the Chinese guidelines for the endovascular treatment of acute ischemic stroke 2015 [11, 12].

### Data collection

Baseline characteristics such as demographics, vascular risk factors, previous use of anti-platelet aggregation drugs, Trial of ORG 10172 in acute stroke treatment (TOAST) types on admission, NIHSS scores on admission, ASPECTS on admission, systolic BP (SBP) and diastolic BP (DBP) on admission, laboratory values, and type of treatment for the EVT were collected. The degree of recanalization at the end of EVT was measured using the Thrombolysis in Cerebral Infarction (TICI) score [13] as obtained from the reports of interventional specialists (C.W. and F.Z.). All patients were examined via brain CT in the first 24 h after EVT to determine any changes in intracranial haemorrhage using the criteria developed by the European Cooperative Acute Stroke Study (ECASS) [14]: HI1, small petechiae with an indistinct border within the vascular territory; HI2, more confluent petechiae, no mass effect; PHI, hematoma within infarcted tissue, occupying < 30% of the infarcted area, no substantive mass effect; and PH2, > 30% of the infarcted area with significant space-occupying effect or parenchymal hematoma distant from the infarcted brain tissue.

### BP monitoring and BPV presentation post EVT

The hourly SBP and DBP of all patients were recorded during the first 24 h following EVT. Postoperative management of blood pressure depended on whether the responsible vessels were successfully recanalized according to the Chinese guidelines for the endovascular treatment of acute ischaemic stroke 2015 [12], which recommend a target BP of 20–30 mmHg lower than that at baseline, but it should not be less than 90/60 mmHg in patients with successful recanalization. For patients without successful recanalization, permissive hypertension was set at a systolic blood pressure more than 150 but not exceeding 180 mmHg. For patients pretreated with intravenous thrombolysis, permissive hypertension was set at < 180/105 mmHg. All patients with postoperative hypertension were treated with intravenous urapidil (first choice) or sodium nitroprusside (second choice) when BP levels exceeded the former prespecified cut-offs. We documented the maximum, minimum, and mean arterial BP (MAP, [SBP + 2 × DBP]/3) levels for each individual. Based on previously published studies, BPV was calculated using the following equation:

(1) Standard deviation of mean BP (SD): $\sqrt{\left(1/\left(n-1\right){\sum}_{\left(i=1\right)}^{\left(n-1\right)}{\left({\mathrm{BP}}_i-{\mathrm{BP}}_{mean}\right)}^2\right)}$,

(2) Coefficient of variability (CV [%]): SD/BP_mean_ × 100,

(3) Successive variation (SV): $\sqrt{\left(1/\left(n-1\right){\sum}_{\left(i=1\right)}^{\left(n-1\right)}{\left({\mathrm{BP}}_{i+1}-{\mathrm{BP}}_i\right)}^2\right)}$ [15].

### Evaluation of functional prognosis

Functional outcome was evaluated at 3 months by certified neurologists using the mRS score. The patients were then divided into two groups based on the functional outcome score: the favorable and unfavorable outcome groups comprised patients with mRS 0–2 and mRS 3–5, respectively. The mRS scores were determined based on the follow-up findings.

### Statistical analysis

All data analyses were performed using the SPSS V. 25.0 software. Continuous variables were expressed as means±SD (normal distribution) or median with interquartile range (IQR) (skewed distribution). Comparisons between groups were conducted using the Students t-test, Mann-Whitney U test, or χ^2^ test, or One-way ANOVA analysis as appropriate. Univariable and multivariable logistic regression models were used to explore the association between BPV indices during the first 24 h post EVT with 3-month functional outcome before and after adjustment for the following potential confounders: age, sex, hypertension, coronary heart disease, atrial fibrillation, diabetes mellitus, smoking, admission NIHSS scores, admission serum glucose and LDL-C levels, admission SBP and DBP levels, type of anesthesia (general anesthesia vs conscious sedation), baseline ASPECTS, onset to groin puncture time, vascular lesion (M1 of the middle cerebral artery [MCA] vs ICA), frequency of MT, type of EVT and rates of successful recanalization. In the initial univariable analyses, a *P* value < 0.05 was set as the threshold for inclusion in the multivariable models. Odds ratios (OR) and 95% confidence interval (CI) were calculated to determine any associations.

To determine the predictive capabilities according to SBP SV, the receiver operating characteristic (ROC) curves were generated, and the sensitivities, specificities, positive predictive values (PPV) and negative predictive values (NPV) of systolic SV were calculated. Because the interaction between BPV and successful reperfusion or offending artery was significant, a subgroup analysis by BPV parameters with 3-month functional outcome were used. We also examined the impact of BPV on functional outcome based on different systolic SV. Patients were stratified according to the quartile of their systolic SV during the first 24 h post EVT and the distribution of the patients with favorable outcomes was calculated in each group.

## Results

### Patient demographics and clinical characteristics

Among 83 patients who underwent emergency EVT in our stroke unit, 11 (13.3%) patients were excluded owing to the following causes: 2 (2.4%) died as a result of non-cardiovascular disease, 4 (4.8%) had inadequate BP during the first 24 h, 3 (3.6%) exited the study during the 3-month follow-up, and 2 (2.4%) died as a result of cerebral hernia. As a result, 72 patients with AIS-LVO within the anterior circulation were enrolled in this study.

The baseline clinical demographic characteristics of the study population are summarized in Table 1. Of the 72 patients, including 42 (58.3%) with favorable outcomes and 30 (41.7%) with unfavorable outcomes at 3-months, the mean age was 64.8 ± 10.9 years, and 27 (37.5%) were women. The median NIHSS score at admission was 14 points [IQR, 9–19], while the median ASPECTS was 8 points [IQR, 7–9]. Of the 72 patients, 86.1% patients achieved recanalization (TICI 2b or 3). In total, 26.4% patients received combined intravenous thrombolysis and thrombectomy, 13.9% of patients were treated with intra-arterial thrombolysis alone, and 59.7% of patients were treated with direct mechanical thrombectomy. Intracranial haemorrhagic transformation occurred in 13 patients (18.0%), while the hemorrhagic transformation was no different between the patients with the three treatments of EVT (Additional file 1).

**Table 1:** Baseline characteristics of patients in the two outcome groups

Variable	Total	Favorable outcome group (*n* = 42, 58.3%)	Unfavorable outcome group (*n* = 30, 41.7%)	*P* value
Age (years), mean ± SD	64.8 ± 10.9	64.5 ± 11.8	65.1 ± 9.8	0.820
Male, n (%)	45 (62.5)	27 (64.3)	18 (60.0)	0.711
Hypertension, n (%)	48 (66.7)	28 (66.7)	20 (66.7)	1.000
Diabetes mellitus, n (%)	23 (31.9)	17 (40.5)	6 (20.0)	0.066
Coronary heart disease, n (%)	21 (29.2)	12 (28.6)	9 (30.0)	0.895
Atrial fibrillation, n (%)	24 (33.3)	16 (31.8)	8 (26.7)	0.310
Previous history of cerebrovascular disease, n (%)	10 (13.9)	5 (11.9)	5 (16.7)	0.565
Previous antiplatelet therapy, n (%)	14 (19.4)	9 (21.4)	5 (16.7)	0.615
Current smoker, n (%)	34 (47.2)	21 (50.0)	13 (43.3)	0.576
NIHSS score at admission, median (IQR)	14 (9–19)	13 (8–17)	17 (12–20)	0.015^a^
Glucose level at admission (mg/dL), mean ± SD	152.3 ± 85.0	163.8 ± 108.0	135.0 ± 30.6	0.157
SBP level at admission (mmHg), mean ± SD	153.8 ± 23.5	146.9 ± 18.5	163.5 ± 25.5	0.003^a^
DBP level at admission (mmHg), mean ± SD	85.2 ± 13.4	83.4 ± 12.8	87.6 ± 14.0	0.189
LDL-C at admission (mg/dL), median (IQR)	44.73 (34.97–55.71)	45.18 (34.43–53.15)	44.01 (35.19–57.60)	0.541
TOAST type, n (%)				
Large artery atherosclerosis	44 (61.1)	23 (54.8)	21 (70.0)	0.442
Cardioembolism	23 (31.9)	16 (38.1)	7 (23.3)	
Clear reason	4 (5.6)	2 (4.8)	2 (6.7)	
Unknown reason	1 (1.4)	1 (2.4)	0 (0.0)	
ASPECTS at admission, median (IQR)	8 (7–9)	8 (8-9)	7 (6.75–8)	< 0.001^a^
Vascular lesion				
M1 of the middle cerebral artery	51 (70.8)	35 (83.3)	16 (53.3)	0.006^a^
Internal carotid artery	21 (29.2)	7 (16.7)	14 (46.7)	
Type of anesthesia				
General anesthesia, n (%)	9 (12.5)	4 (9.5)	5 (16.7)	0.366
Conscious sedation, n (%)	63 (87.5)	38 (90.5)	25 (83.3)	0.366
Time from stroke onset to groin puncture (min), mean ± SD	290.5 ± 80.5	297.0 ± 72.5	281.4 ± 91.1	0.421
Type of endovascular treatment				
Combined intravenous thrombolysis and thrombectomy, n (%)	19 (26.4)	8 (19.0)	11 (36.7)	0.094
Intra-arterial thrombolysis, n (%)	10 (13.9)	9 (21.4)	1 (3.3)	0.029^a^
Direct mechanical thrombectomy, n (%)	43 (59.7)	25 (59.5)	18 (60.0)	0.968
Frequency of mechanical thrombectomy, median (IQR)	2 (2–3)	2 (1–3)	3 (2–3)	0.024^a^
Rates of successful recanalization, n (%)	62 (86.1)	40 (95.2)	22 (73.3)	0.008^a^
Intracranial haemorrhagic transformation, n (%)				
HI1	5 (6.9)	4 (9.5)	1 (3.3)	0.197
HI2	5 (6.9)	2 (4.8)	3 (10.0)	
PH1	2 (2.8)	0 (0.0)	2 (6.7)	
PH2	1 (1.4)	0 (0.0)	1 (3.3)	

*NIHSS* National Institutes of Health Stroke Scale, *SBP* systolic blood pressure, *DBP* diastolic blood pressure, *LDL-C* low-density lipoprotein cholesterol, *ASPECTS* Alberta Stroke Program Early CT Score, *HI* petechial infarction without space-occupying effect, *PH* haemorrhage (coagulum) with mass effect

^a^Statistically significant

Compared to patients with an unfavorable outcome group, the NIHSS scores, admission SBP level, and frequency of MT were significantly lower in the favorable outcome group (all *P* < 0.05). Patients with a 3-month favorable outcome were more likely to have lesions in the M1 of middle cerebral artery, to have higher rates of successful recanalization, to have higher admission ASPECT scores, and to receive intra-arterial thrombolysis alone. The rates of vascular risk factors, time of symptom onset to groin puncture, and HI were not significantly different between the two groups.

Table 2 lists the baseline characteristics of patients in different groups, in which patients are divided into four groups according to systolic SV values quartiles: Systolic SV values ≤10.96, 10.97–12.71, 12.72–14.24, and>14.24. The frequency of MT, ASPECT score at admission and rates of successful recanalization post EVT differed among the four groups (all *P* < 0.05).

**Table 2:** Baseline characteristics of patients grouped by systolic SV quartile

Variable	Quartile 1 (≤10.96) *n* = 18	Quartile 2 (10.97–12.71) *n* = 18	Quartile 3 (12.72–14.24) *n* = 18	Quartile 4 (>14.24) *n* = 18	*P*-value
Age (years), mean ± SD	64.8 ± 10.2	60.1 ± 12.5	64.6 ± 10.3	69.4 ± 9.4	0.090
Male, n (%)	11 (61.1)	12 (66.7)	9 (50.0)	13 (72.2)	0.557
Hypertension, n (%)	10 (55.6)	12 (66.7)	12 (66.7)	14 (77.8)	0.572
Diabetes mellitus, n (%)	5 (27.8)	9 (50.0)	4 (22.2)	5 (27.8)	0.287
Coronary heart disease, n (%)	3 (16.7)	7 (38.9)	7 (38.9)	4 (22.2)	0.330
Atrial fibrillation, n (%)	7 (38.9)	6 (33.3)	5 (27.8)	6 (33.3)	0.919
Previous history of cerebrovascular disease, n (%)	3 (16.7)	3 (16.7)	0 (0.0)	4 (22.2)	0.243
Current smoker, n (%)	8 (44.4)	12 (66.7)	5 (27.8)	9 (50.0)	0.134
NIHSS score at admission, median (IQR)	9.5 (7.8–14.8)	13 (9.0–16.3)	18 (8.5–21.3)	16.5 (13.8–20.0)	0.070
Glucose level at admission (mg/dL), mean ± SD	149.0 ± 78.1	184.3 ± 141.3	138.4 ± 37.2	137.4 ± 33.7	0.309
SBP level at admission (mmHg), mean ± SD	143.4 ± 23.9	156.7 ± 17.3	158.7 ± 27.0	156.4 ± 23.4	0.189
DBP level at admission (mmHg), mean ± SD	81.9 ± 15.1	87.3 ± 11.1	82.7 ± 12.9	88.8 ± 14.0	0.335
LDL-C at admission (mg/dL), median (IQR)	43.5 (33.9–56.4)	49.0 (36.2–56.0)	36.8 (30.3–3.56.7)	46.2 (41.5–56.4)	0.384
TOAST type, n (%)					
Large artery atherosclerosis	9 (50.0)	11 (61.1)	12 (66.7)	12 (66.7)	0.442
Cardioembolism	6 (33.3)	6 (33.3)	5 (27.8)	6 (33.3)	0.377
Clear reason	3 (16.7)	0 (0.0)	1 (5.6)	0 (0.0)	
Unknown reason	0 (0.0)	1 (5.6)	0 (0.0)	0 (0.0)	
ASPECTS at admission, median (IQR)	7.8 (8–9)	8 (8–9)	7 (7–8.3)	7 (6–7.3)	< 0.001^a^
Vascular lesion					
M1 of the middle cerebral artery	14 (77.8)	16 (88.9)	10 (55.6)	11 (61.1)	0.106
Internal carotid artery	4 (22.2)	2 (11.1)	8 (44.4)	7 (38.9)	
Type of anesthesia					
Conscious sedation, n (%)	17 (94.4)	14 (77.8)	16 (88.9)	16 (88.9)	0.491
General anesthesia, n (%)	1 (5.6)	4 (22.2)	2 (11.1)	2 (11.1)	
Time from stroke onset to groin puncture (min), mean ± SD	284.6 ± 60.7	300.1 ± 75.8	308.7 ± 84.6	268.7 ± 97.4	0.467
Type of endovascular treatment					
Combined intravenous thrombolysis and thrombectomy, n (%)	4 (22.2)	7 (38.9)	4 (22.2)	4 (22.2)	0.777
Intra-arterial thrombolysis alone, n (%)	4 (22.2)	2 (11.1)	2 (11.1)	2 (11.1)	
Direct mechanical thrombectomy, n (%)	10 (55.6)	9 (50.0)	12 (66.7)	12 (66.7)	
Frequency of mechanical thrombectomy, median (IQR)	1.5 (0–3)	1.5 (1–2.3)	2 (1.8–3)	2.5 (2–3.3)	0.038^a^
Rates of successful recanalization, n (%)	18 (100.0)	17 (94.4)	14 (77.8)	13 (72.2)	0.048^a^
Intracranial haemorrhagic transformation, n (%)					
HI1	1 (5.6)	1 (5.6)	1 (5.6)	2 (11.1)	0.731
HI2	2 (11.1)	0 (0.0)	1 (5.6)	2 (11.1)	
PH1	0 (0.0)	0 (0.0)	1 (5.6)	1 (5.6)	
PH2	0 (0.0)	0 (0.0)	0 (0.0)	1 (5.6)	

*NIHSS* National Institutes of Health Stroke Scale, *SBP* systolic blood pressure, *DBP* diastolic blood pressure, *LDL-C* low-density lipoprotein cholesterol, *ASPECTS* Alberta Stroke Program Early CT Score, *HI* petechial infarction without space-occupying effect, *PH* haemorrhage (coagulum) with mass effect

^a^Statistically significant

### BPV and 3-month functional outcome

**Fig. 1:**
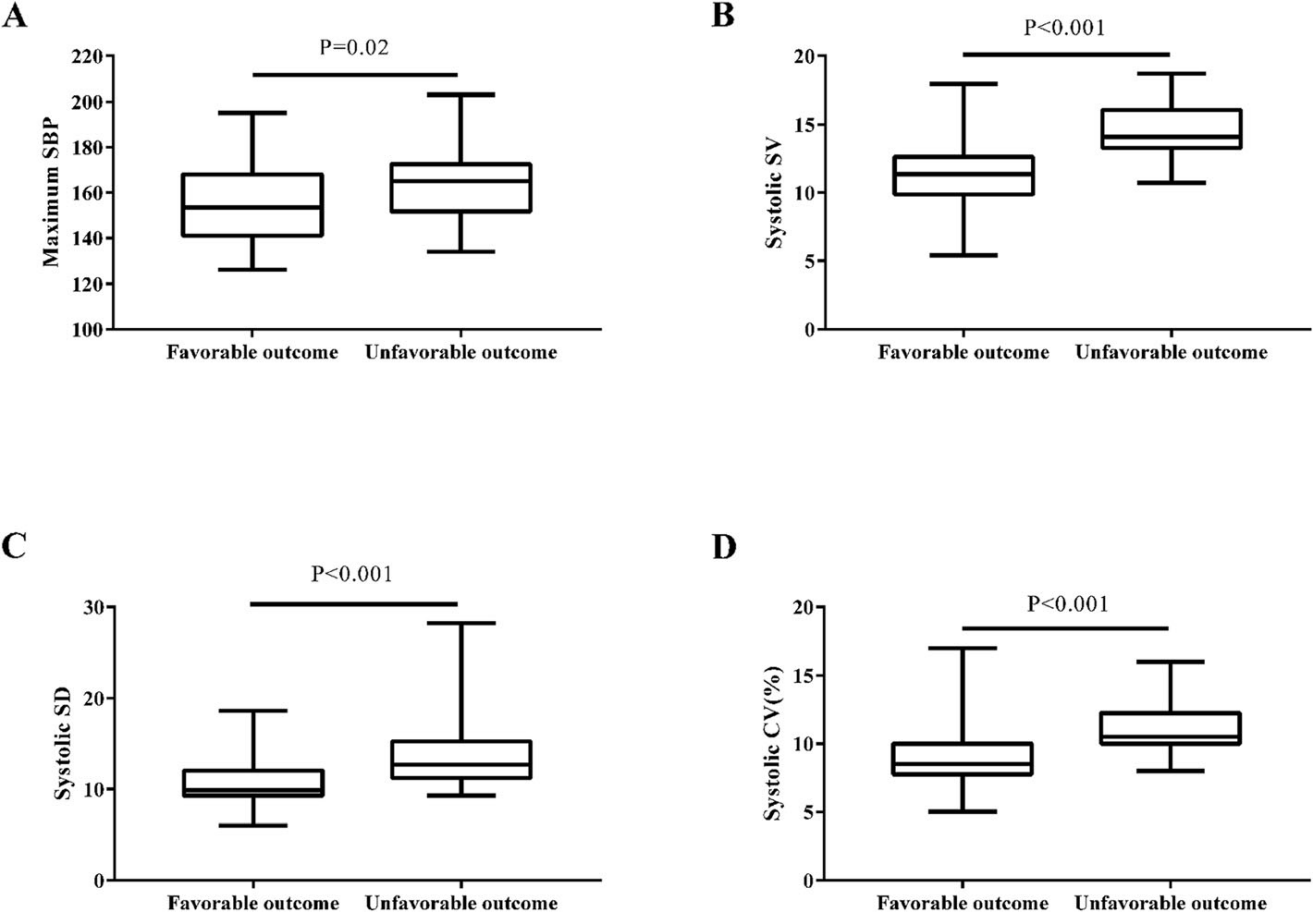
Maximum SBP (**a**) and systolic SV (**b**), SD (**c**), CV (**d**) within 24 h post-EVT in the two groups. *EVT* endovascular treatment; *SBP* systolic blood pressure; *SV* successive variation; *SD* standard deviation; *CV* coefficient of variation

In this study (Fig. 1), we detected the difference in maximum SBP, systolic CV, SV, and SD between the two outcome groups. Patients with unfavorable prognosis had higher maximum SBP ([163.5 ± 15.6] vs. [154.3 ± 16.8], *P* = 0.02), systolic CV ([11.0% ± 1.8%] vs. [8.8% ± 2.0%], *P* < 0.001), SV ([14.6 ± 2.0] vs. [11. 4 ± 2.3], *P* < 0.001), and SD ([13.8 ± 3.9] vs. [10. 5 ± 2.4], *P* < 0.001). We found no significant difference in the level of MAP, mean SBP, minimum SBP, and dates of DBP variability between the two groups (*P* > 0.05). On subgroup analysis, we also found the maximum SBP, systolic SV, CV, SD were lower in patients with a favorable outcome in the successful recanalization group; however no significant difference was observed in the non-successful recanalization group. Lower systolic SV, CV, and SD were found in M1 of the MCA lesion group, according to vascular lesions. In the ICA lesion group, the systolic SV was lower amongst those patients with a favorable outcome; other BPV parameters were not found to be different (Table 3).

**Table 3:** Comparison of SBP and DBP variability parameters between different subgroups

Successful recanalization (*n* = 62, 86.1%)				Non-successful recanalization (*n* = 10, 13.9%)			
BPV index	Favorable outcome	Unfavorable outcome	*P* value	BPV index	Favorable outcome	Unfavorable outcome	*P* value
Maximum SBP	153.4 ± 15.9	165.4 ± 16.0	0.006	Maximum SBP	172.0 ± 32.5	158.1 ± 13.9	0.341
Maximum DBP	94.8 ± 9.6	90.4 ± 6.8	0.063	Maximum DBP	106.5 ± 7.8	94.9 ± 9.6	0.154
Minimum SBP	111.2 ± 14.8	110.0 ± 13.7	0.751	Minimum SBP	131.0 ± 38.2	103.9 ± 12.8	0.496
Minimum DBP	60.0 ± 10.3	58.3 ± 9.3	0.498	Minimum DBP	64.5 ± 30.4	59.4 ± 8.5	0.851
Mean SBP	130.9 ± 15.0	134.2 ± 13.7	0.395	Mean SBP	151.7 ± 33.3	131.1 ± 18.0	0.239
Mean DBP	76.1 ± 8.9	73.7 ± 8.0	0.296	Mean DBP	77.4 ± 25.5	77.1 ± 7.5	0.987
Systolic SV	11.4 ± 2.3	14.4 ± 2.0	< 0.001	Systolic SV	12.8 ± 1.8	15.0 ± 1.9	0.174
Systolic SD	10.0 ± 2.4	14.0 ± 4.3	< 0.001	Systolic SD	8.7 ± 1.6	13.2 ± 2.6	0.052
Systolic CV	(8.8 ± 2.0)%	(10.8 ± 1.7)%	< 0.001	Systolic CV	(8.8 ± 3.1)%	(11.7 ± 2.2)%	0.160
Diastolic SV	9.8 ± 3.2	8.8 ± 2.3	0.182	Diastolic SV	12.1 ± 5.0	10.4 ± 3.2	0.544
Diastolic SD	8.3 ± 2.2	8.0 ± 2.0	0.610	Diastolic SD	8.7 ± 3.7	8.2 ± 1.7	0.796
Diastolic CV	(13.1 ± 4.8)%	(12.1 ± 3.6)%	0.377	Diastolic CV	(17.7 ± 12.3)%	(13.8 ± 5.0)%	0.459
M1 of MCA				ICA			
BPV index	Favorable outcome	Unfavorable outcome	*P* value	BPV index	Favorable outcome	Unfavorable outcome	*P* value
Maximum SBP	154.2 ± 16.6	159.5 ± 14.9	0.278	Maximum SBP	154.7 ± 19.5	168.0 ± 15.5	0.106
Maximum DBP	96.6 ± 8.8	90.8 ± 8.6	0.032	Maximum DBP	89.1 ± 13.0	92.5 ± 6.6	0.437
Minimum SBP	112.9 ± 16.1	109.1 ± 15.0	0.420	Minimum SBP	108.0 ± 17.3	107.5 ± 12.0	0.939
Minimum DBP	60.0 ± 11.3	57.6 ± 8.1	0.450	Minimum DBP	61.6 ± 11.0	59.6 ± 10.0	0.691
Mean SBP	132.1 ± 16.0	133.1 ± 16.6	0.847	Mean SBP	130.9 ± 18.5	133.8 ± 12.9	0.678
Mean DBP	76.1 ± 9.4	74.4 ± 7.4	0.512	Mean DBP	76.4 ± 11.0	74.9 ± 8.8	0.734
Systolic SV	11.5 ± 1.9	14.5 ± 1.9	< 0.001	Systolic SV	11.4 ± 3.9	14.6 ± 2.1	0.02
Systolic SD	10.1 ± 2.0	12.6 ± 2.6	< 0.001	Systolic SD	12.8 ± 3.3	15.1 ± 4.7	0.259
Systolic CV	(8.8 ± 1.9)%	(11.0 ± 2.1)%	< 0.001	Systolic CV	(8.7 ± 2.5)%	(11.0 ± 1.6)%	0.018
Diastolic SV	10.4 ± 3.3	9.8 ± 2.9	0.533	Diastolic SV	7.7 ± 2.1	8.5 ± 2.3	0.406
Diastolic SD	8.5 ± 2.3	8.3 ± 1.9	0.709	Diastolic SD	7.4 ± 1.3	7.9 ± 2.0	0.552
Diastolic CV	(14.0 ± 5.3)%	(13.3 ± 4.2)%	0.644	Diastolic CV	(10.0 ± 2.0)%	(11.6 ± 3.8)%	0.288

*SBP* Systolic blood pressure, *DBP* Diastolic blood pressure, *BPV* Blood pressure variability, *SD* Standard deviation, *CV* Coefficient of variation, *SV* Successive variation

### Influencing factors of 3-month functional independence

Table 4 summarizes the univariable and multivariable associations of BP measurements after EVT and other clinical characteristics with the 3-month functional prognosis. The following variables were found to be significantly related (*P* < 0.05) to 3-month functional independence in the initial univariable analysis: NIHSS score at admission, SBP at admission, ASPECTS at admission, M1of the MCA occlusion, frequency of mechanical thrombectomy, measurement of EVT, successful recanalization, maximum SBP and systolic SD, CV, and SV post MT. After adjusting for potential confounders, multivariable logistic regression revealed that systolic SV (OR: 4.273, 95% CI: 1.030 to 17.727, *P* = 0.045) was an independent predictor of unfavorable outcome, and a high ASPECTS was independently associated with a better likelihood of a favorable outcome (OR: 0.200, 95% CI: 0.054 to 0.744, *P* = 0.016).

**Table 4:** Univariate and multivariate analysis of the favorable outcomes after EVT

Variable	Univariable logistic regression analysis		Multivariable logistic regression analysis	
	OR (95% CI)	*P* value^*^	OR (95% CI)	*P* value
Age	1.005 (0.963–1.050)	0.817		
Male	1.200 (0.457–3.151)	0.711		
Hypertension	1.000 (0.370–2.702)	1.000		
Coronary heart disease	1.071 (0.383–2.997)	0.895		
Atrial fibrillation	0.591 (0.213–1.641)	0.313		
Diabetes mellitus	0.368 (0.124–1.089)	0.071		
Smoking	0.765 (0.298–1.962)	0.577		
Glucose level at admission	0.893 (0.755–1.057)	0.189		
NIHSS at admission	1.072 (1.002–1.148)	0.045	0.931 (0.808–1.073)	0.325
SBP level at admission	1.036 (1.010–1.063)	0.006	1.045 (0.993–1.100)	0.092
DBP level at admission	1.025 (0.988–1.063)	0.189		
LDL-C at admission	1.170 (0.724–1.891)	0.521		
Conscious sedation	1.900 (0.465–7.769)	0.372		
ASPECTS at admission	0.268 (0.138–0.522)	< 0.001	0.200 (0.054–0.744)	0.016
Time from stroke onset to groin puncture	0.998 (0.992–1.003)	0.415		
M1of the MCA occlusion	0.229 (0.077–0.675)	0.008	0.076 (0.005–1.078)	0.057
Frequency of mechanical thrombectomy	0.098 (0.011–0.860)	0.036	1.499 (0.038–59.877)	0.830
Combined intravenous thrombolysis and thrombectomy	2.461 (0.844–7.172)	0.099		
Intra-arterial thrombolysis	0.097 (0.012–0.801)	0.030	0.012 (0.000–1.457)	0.071
Successful recanalization	0.138 (0.027–0.075)	0.017	0.030 (0.001–1.842)	0.095
Maximum SBP post EVT^a^	1.036 (1.004–1.069)	0.803	0.894 (0.777–1.028)	0.116
Maximum DBP post EVT^a^	0.953 (0.901–1.008)	0.091		
Minimum SBP post EVT^a^	0.983 (0.952–1.015)	0.297		
Minimum DBP post EVT^a^	0.983 (0.939–1.030)	0.482		
Mean SBP post EVT^a^	1.006 (0.976–1.037)	0.686		
Mean DBP post EVT^a^	0.980 (0.929–1.034)	0.456		
Systolic SD post EVT^a^	1.531 (1.203–1.948)	0.001	1.217 (0.803–1.842)	0.355
Systolic CV post EVT^a^	2.732E+ 28 (8.024E+ 12–9.303E+ 43)	< 0.001	0.000 (0.000–7.704E+ 24)	0.221
Systolic SV post EVT^a^	2.046 (1.444–2.898)	< 0.001	4.273 (1.030–17.727)	0.045

*NIHSS* National Institutes of Health Stroke Scale, *SBP* Systolic blood pressure, *ASPECTS* Alberta Stroke Program Early CT Score, *MCA* Middle cerebral artery, *EVT* Endovascular treatment; *LDL-C* Low-density lipoprotein cholesterol; *DBP* Diastolic blood pressure; *SD* Standard deviation; *CV* Coefficient of variation; *SV* Successive variation

*Cut-off of *P* < 0.05 was used for selection of candidate variables for inclusion in multivariable logistic regression models

^a^During the 24 h following the endovascular treatment

### mRS score distribution according to quartiles of systolic SV

**Fig. 2:**
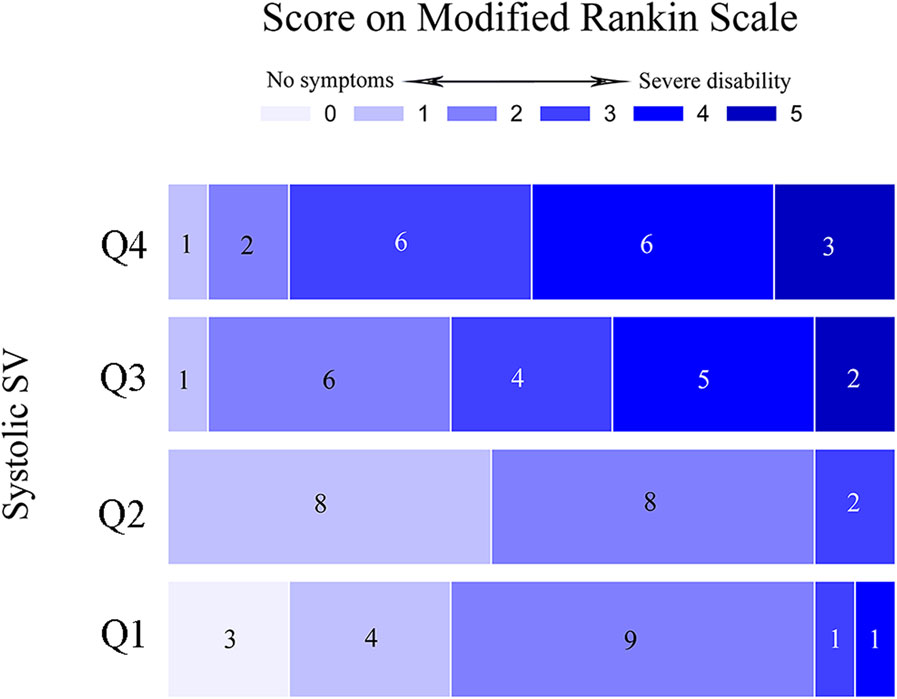
Modified Rankin Scale scores distribution according to quartiles of systolic SV

Patients were divided into 4 groups according to systolic SV values by quartile to clarify the relationship between SV values and mRS scores (Fig. 2). One-way ANOVA analysis demonstrated that there was a significant in mRS scores at 3-month of the four groups (*P* < 0.001). In addition, Multiple Post Hoc Comparisons showed compared with the group with high systolic SV, those with lower systolic SV had lower mRS scores at 3 months (*P* < 0.001, OR = − 1.833, 95% CI = − 2.722 to − 0.945 for Q1:Q4; *P* < 0.001, OR = − 1.444, 95% CI = − 2.333 to − 0.556 for Q1:Q3; *P* < 0.001, OR = − 1.389, 95% CI = − 2.277 to − 0.500 for Q2:Q3; *P* < 0.001, OR = − 1.778, 95% CI = − 2.666 to − 0.889 for Q2:Q4;), no statistically difference in mRS scores was noted in patients with systolic SV Q1 and Q2 (*P* = 0.998), Q3 and Q4(*P* = 0.659). Furthermore, after adjusted for age, sex, frequency of MT, ASPECT score at admission and rates of successful recanalization, the risk of unfavorable outcome at 3-month was significantly decreased in patients with low systolic SV levels compared with the group with high systolic SV levels (*P* = 0.015, OR = 0.056, 95% CI =0.007 to 0.433 for Q1:Q4). After additional adjustment for hypertension, diabetes mellitus, coronary heart disease, atrial fibrillation, smoking, admission NIHSS scores, admission serum glucose, LDL-C, admission SBP, admission DBP, onset to groin puncture time, vascular lesion and type of EVT, the significance persisted (*P* = 0.004, OR = 0.008, 95% CI =0.000 to 0.141 for Q1:Q4).

### ROC analysis

**Fig. 3:**
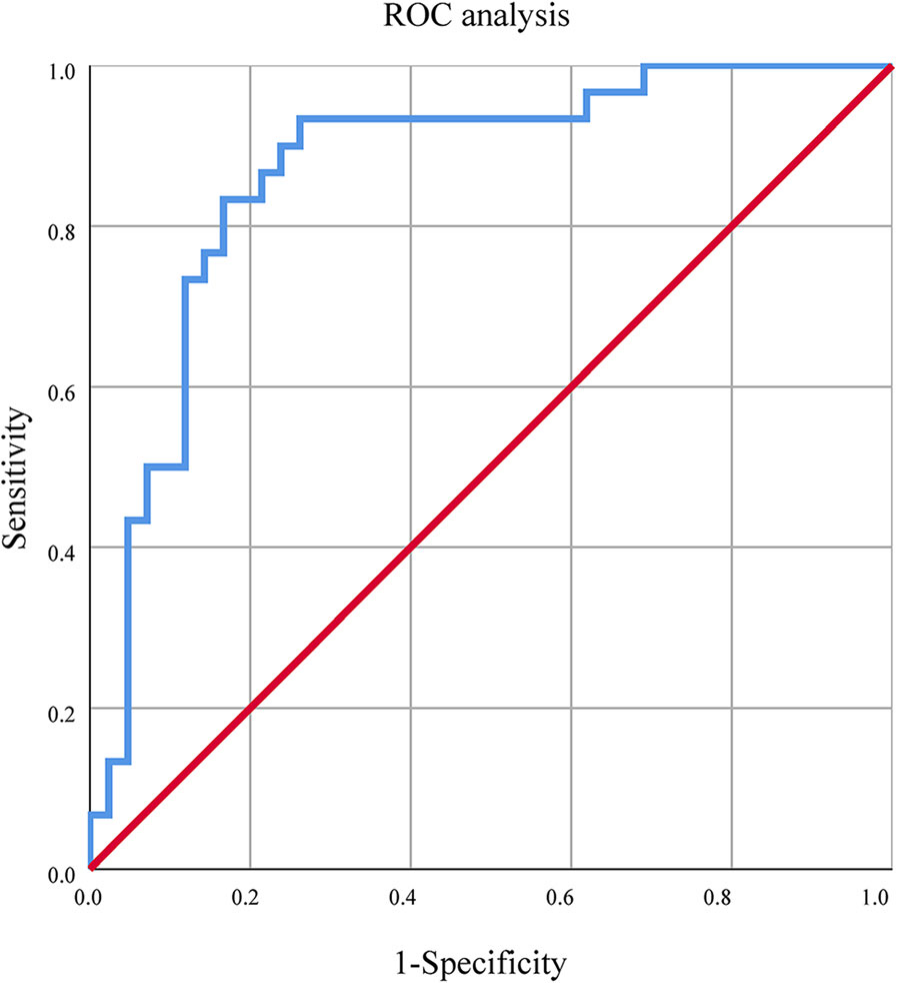
Systolic SV and 3-month unfavorable outcomes. *SBP* systolic blood pressure; *SV* successive variation

ROC analysis demonstrated that the areas under the curve (AUC) of systolic SV for predicting unfavorable outcome was 0.868 (95% CI: 0.781 to 0.955, *P* < 0.001; Fig. 3). The optimal cut-off value was 12.499, which resulted in 93.3% sensitivity, 73.8% specificity, 71.1% PPV, and 91.2% NPV (Table 5). This indicates that an systolic SV of 12.499 had an excellent predictive value for a poor 3-month functional outcome.

**Table 5:** Cut-off values of systolic SV

Values	Best cut-off	Sensitivity (%)	Specificity (%)	PPV^*^(%)	NPV^*^(%)
Systolic SV	12.499	93.3	73.8	71.1	91.2

*SV* Successive variation; *PPV* Positive predictive values; *NPV* Negative predictive values

## Discussion

The clinical outcome in patients with ischaemic stroke is affected by many factors, including age, severity of stroke, collateral compensation, time of successful reperfusion, and device selected for EVT. BP management and its effect on functional outcome is particularly controversial. A previous study showed that increased systolic BPV positively contributed to symptomatic intracerebral haemorrhage and death after intravenous thrombolysis [16]. However, less is known about the effect of short-term BPV after EVT on the early outcomes of AIS-LVO patients. Our study shows that lower maximum SBP and systolic CV, SV, and SD levels during the first 24 h after EVT are related to a better 3-month functional outcome, which was consistent with the results reported by Bennett [17].

BPV is divided into physiological and pathological variability, which fluctuates with physiological regulation, environmental changes, and pathological influence. The possible pathophysiological mechanisms regarding short-term BPV in AIS patients with EVT are not clear. In a previous study, increased BPV may promote shear force of blood vessels and produce vascular inflammation by increasing endothelial expression of cytokines, which affect the structure of the vascular wall and lead to the formation of atherosclerotic plaques [10, 18]. Another hypothesis is that the effects of increased BPV on brain tissue may vary with the degree of impaired cerebral autoregulation [19], and the cerebral blood flow dependent on cerebral perfusion pressure and blood viscosity [20]. Endovascular therapy can not only stimulate endothelial cells, but also change the cerebral perfusion pressure and vascular resistance in LVO-AIS patients. Therefore, there is a potential correlation between blood pressure variability and outcome in LVO-AIS patients after intra-arterial treatment.

BPV are commonly quantified by calculating SD, CV, and SV [10]. Several studies have shown that higher systolic CV or SD is associated with poor prognosis after stroke [21, 22]; however, systolic SV, an indicator of systolic blood pressure variability, is more commonly used in many studies, because it can better reflect the time-series variability of BP, while other parameters, such as SD and CV, ignore the temporal change of data, resulting in the same SD or CV in individuals with different clinical characteristics [23]. In our study, we confirmed that the systolic SV, rather than systolic CV and SD, was closely associated with 3-month functional outcome. Lower systolic SV levels may be beneficial to achieving 3-month functional independence. After adjusting for various confounders, the correlation remained significant. These findings suggest that maintaining a stable BP may be more useful than merely controlling the BP levels after EVT.

A study of 217 patients who underwent MT showed that a higher maximum SBP was closely related to 3-month mortality and poor outcome. Each 10 mmHg increase in maximum SBP during the first 24 h post MT was associated with a lower 3-month functional prognosis and a higher odds of 3-month mortality [24]. Although our study did not find the maximum SBP to be an influential factor for functional outcome, this may be related to our study subjects’ characteristics. Our research found that the rate of successful recanalization was higher in the favorable outcome group, which also had lower maximum SBP, indirectly suggesting that patients with successful reperfusion are more likely to benefit from lower SBP [25].

Some studies showed that BP within the first 48 h after a stroke showed a U-shaped correlation with clinical outcome [17, 26], particularly in patients with non-recanalization. The authors argued that patients with unsuccessful recanalization had larger infarct size and ischaemic penumbra, and impaired cerebral autoregulation led to further enlargement of the ischaemic penumbra [17]. The effect of BPV on the ischaemic penumbra is different from that on the infarct core [10]. In the hours after onset, potentially ischaemic penumbra are particularly sensitive to blood pressure fluctuations, with sudden drops in blood pressure increasing the risk of tissue ischaemia and reducing the chance of reperfusion, and sudden increases in blood pressure increasing the risk of bleeding. Patients who received EVT and had unsuccessful reperfusion enlarged the ischaemic penumbra, which was sensitive to blood pressure variability, greatly increasing the risk of neurological deterioration caused by BPV. In addition, cerebral ischaemia and MT itself may lead to the destruction of blood-brain barrier, resulting in vasogenic edema and haemorrhagic transformation after infarction. Moreover, iatrogenic injury to endothelial cells during MT can cause a series of reperfusion-related injuries [27] that not only increase intracranial haemorrhage associated with SBP, but also lead to adverse functional prognosis. In this study, the subjects with low systolic SV had a higher rate of successful recanalization, we therefore deduced that successful recanalization is a significant factor for systolic SV. In addition, owing to the small number of cases of patients without recanalization, no significant difference was found between the two groups in terms of functional outcome. Future studies should focus on enlarging the sample size to be more adequately powered for these sorts of subanalyses. Another study also showed that the peak level of SBP was closely related to poor outcome regardless of whether LVO recanalization was achieved or not. The authors suggested that this is probably because abnormally elevated BP may be associated with potential collateral circulation damage [28].

The impairment of cerebral autoregulation is influenced by infarct size [29]. Thus, BPV may exert a greater pathophysiological role in patients with severe stroke than those with mild stroke. In our study, patients with favorable prognosis were more likely to have M1 of the middle cerebral artery affected, which may, in theory, produce a smaller infarct volume. Subgroup analysis in our cohort also confirmed that those with M1 of MCA lesions, the systolic SV, CV, and SD were lower in the favorable outcome group.

Several limitations of the present study need to be acknowledged. First, this was a single-centre retrospective study with a relatively small sample size. Thus, selection bias in baseline data was unavoidable. Second, a recent study demonstrated that BPV post MT may increase the rate of symptomatic intracranial haemorrhage (sICH) [10], but we did not evaluate the relationship between BPV and sICH because the patients who developed intracranial haemorrhage during follow-up were classified according to ECASS criteria without the clinical classification for sICH. Third, variable reasons such as the varying time from stroke onset to arrival at our hospital for the first BP measurement and differences in time intervals between BP measurements may have introduced bias in our results. However, we exerted every effort to provide reliable dates to mitigate the inherent limitations. Fourth, SD, CV and SV are not suitable for the long-term evaluation of BPV after EVT; severe stroke with a poor prognosis may give rise to greater variability in BP. Therefore, additional well-designed and larger prospective randomized cohort studies are required to confirm the association of BPV and functional prognosis and to determine optimal strategies to reduce the BPV.

## Conclusions

Decreased systolic SV following intra-arterial therapies result in favorable 3-month outcomes. Systolic SV may therefore be a novel predictor of functional prognosis in LVO patients.

## Supplementary information

**Additional file 1:Table S1.** Intracerebral haemorrhage of patients post-EVT. (16 kb)

## Data Availability

The datasets used and/or analysed during the current study are available from the corresponding author on reasonable request.

## Abbreviations

AIS: Acute ischaemic stroke
ASPECTS: Alberta Stroke Program Early CT Score
AUC: Area under the curve
BP: Blood pressure
BPV: Blood pressure variability
CI: Confidence interval
CV: Coefficient of variation
DBP: Diastolic blood pressure
ECASS: European Cooperative Acute Stroke Study
EVT: Endovascular treatment
HI: Petechial infarction without space-occupying effect
IQR: Interquartile range
LDL-C: Low-density lipoprotein cholesterol
LVO: Large-vessel occlusion
MCA: middle cerebral artery
mRS: Modified Rankin Scale
MT: Mechanical thrombectomy
NIHSS: National Institutes of Health Stroke Scale
OR: Odds ratio
PH: Haemorrhage (coagulum) with mass effect
ROC: Receiver operating characteristic
SBP: Systolic blood pressure
SD: Standard deviation
sICH: Symptomatic intracerebral haemorrhage
SV: Successive variation
TICI: Thrombolysis in Cerebral Infarction
TOAST: Trial of ORG 10172 in acute stroke treatment
