# Supplementary material for: Effect of short-term blood pressure variability on functional outcome after intra-arterial treatment in acute stroke patients with large-vessel occlusion
Source: BMC Neurol. 2019 Sep 26;19:228. doi: 10.1186/s12883-019-1457-5 (PMC6764143; doi:10.1186/s12883-019-1457-5)
Supplement: Supplementary file 1 — Additional file 1:Table S1. Intracerebral haemorrhage of patients post-EVT. (16 kb) [file 12883_2019_1457_MOESM1_ESM.docx]

**Additional file 1**: **Table S1: Intracerebral haemorrhage of patients post-EVT**

| **Type of treatment** | **Total (%)** | **ICH (%)** | **P** |
| --- | --- | --- | --- |
| Combined intravenous thrombolysis and thrombectomy | 19 (26.4%) | 3 (15.8) | 0.689 |
| Direct intra-arterial thrombolysis | 10 (13.9) | 1 (10) |  |
| Direct mechanical thrombectomy | 43 (59.7) | 9 (20.9) |  |

*ICH* intracerebral haemorrhage; *EVT* Endovascular treatment.
